# Distinct blood volume and left ventricular adaptation to severe obesity in middle‐aged adults at risk for heart failure

**DOI:** 10.1002/ejhf.70037

**Published:** 2025-09-09

**Authors:** Joseph Campain, Denis J. Wakeham, Katrin Dias, James P. MacNamara, Mitchel Samels, Erin J Howden, Graeme Carrick‐Ranson, Michinari Hieda, Benjamin D. Levine, Satyam Sarma, Christopher M. Hearon

**Affiliations:** ^1^ Institute for Exercise and Environmental Medicine Texas Health Presbyterian Hospital Dallas Dallas TX USA; ^2^ Department of Internal Medicine University of Texas Southwestern Medical Center Dallas TX USA; ^3^ Cardiometabolic Health and Exercise Physiology Lab Baker Heart and Diabetes Institute Melbourne VIC Australia; ^4^ Department of Surgery the University of Auckland Auckland New Zealand; ^5^ Department of Applied Clinical Research University of Texas Southwestern Medical Center Dallas TX USA

**Keywords:** Obesity, Blood volume, Ventricular remodelling, Heart failure

## Abstract

**Aims:**

Obesity is commonly hypothesized to lead to the development of heart failure (HF) in part due to increases in blood volume (BV) and left ventricular (LV) remodelling. Whether adiposity and obesity severity are associated with BV expansion and subsequent LV remodelling in middle‐aged individuals at increased risk (IR) prior to the onset of HF is unknown.

**Methods and results:**

We analysed data from 96 middle‐aged (40–64 years) non‐obese (25.8 [23.6–28.6] kg/m^2^) controls (CON) and 126 IR middle‐aged adults (elevated cardiac biomarkers plus established risk factors). IR adults were stratified based upon body mass index class: (1) <30 kg/m^2^, IR_Non‐Obese_ (*n* = 28, 28.2 [24.6–29.9] kg/m^2^); (2) Class I >30–35 kg/m^2^, IR_Class‐I_ (*n* = 39, 33 [31.9–33.6] kg/m^2^); and, (3) Class II/III >35 kg/m^2^, IR_Class‐II/IIII_ (*n* = 59, 41.2 [37.1–43.8] kg/m^2^). BV (carbon monoxide rebreathing), body composition (hydrodensitometry or dual‐energy X‐ray absorptiometry), and LV structure and function (echocardiography) were assessed. Fat mass was independently associated with BV (*β* = 0.17, *p* < 0.001) which was independently associated with LV end‐diastolic volume (LVEDV) index (*β* = 0.54, *p* < 0.001). BV was lower in CON (5046 ± 1123 ml) than all IR groups (IR_Non‐Obese_: 5622 ± 1137; IR_Class‐I_: 6033 ± 1237; IR_Class‐II/III_: 6548 ± 1153 mL; all *p* < 0.05). IR_Class‐II/III_ had greater erythrocyte volume compared to CON (*p* < 0.005), even after normalization to fat‐free mass (CON: 36.2 ± 4.6; IR_Class‐II/III_: 39.9 ± 5.1 ml/kg fat‐free mass; *p* < 0.001). Only IR_Class‐II/III_ had an enlarged LV end‐diastolic volume when normalized to body surface area compared to both CON and IR_Non‐Obese_ (both, *p* < 0.05).

**Conclusions:**

While lean mass is the primary determinant of BV, fat mass is independently associated with BV expansion and larger LVEDV. IR adults with class II/III obesity display distinct LV enlargement that is disproportionate to body size (i.e. LVEDV index) and may represent a physiologically distinct subgroup of obesity as opposed to a simple continuum of disease severity.

## Introduction

Obesity is a strong risk factor for the development of heart failure (HF),^1^ and specifically HF with preserved ejection fraction (HFpEF).^2^, ^3^, ^4^ While the connection between obesity and HFpEF is well established,^5^ many of the largest clinical trials characterizing the pathophysiology and treatment of HFpEF have under‐enrolled patients with severe obesity (Class II/III).^6^ While more recent clinical trials in HFpEF have seen an increase in the mean cohort body mass index (BMI), the beneficial cardiovascular effects of some modern obesity therapies under investigation (sodium–glucose co‐transporter 2 inhibitors, glucagon‐like peptide‐1 agonists) may be driven primarily by improvements in overweight/Class I obesity, and diminished or entirely absent in Class II/III obesity.^7^, ^8^, ^9^ As the prevalence of severe obesity continues to increase, with an estimated one in four adults in the United States expected to have Class II/III obesity by 2030,^10^ data describing the unique pathophysiology of Class II/III obesity are needed to fully understand the cardiovascular adaptations that lead to the obese HF phenotype.^11^

It is commonly hypothesized that the link between chronic obesity and HF is a pathophysiological increase in adiposity that leads to an increase in blood volume (BV), cardiac filling pressure, ventricular size, and stiffness via adipose‐mediated inflammation.^12^ Patients with obesity and established HFpEF have greater estimates of absolute BV, however these estimates have limited reliability in HF.^13^ Nevertheless, estimated BV is associated with cardiac enlargement in HF,^11^ but the relationship between obesity class, adiposity, and measurements of BV and left ventricular (LV) size prior to the onset of HF are not well defined. Because BV and LV size increase with body size, and most directly lean mass,^14^ an independent link between adiposity and BV expansion has not been identified in adults with severe obesity.^14^, ^15^ Therefore, whether adiposity is independently associated with BV expansion and increased LV chamber size in middle‐aged adults at increased risk (IR) for future HF, and the effect of obesity class on these relationships, is unknown.

We assessed the relationship between body composition (body mass, fat‐free mass [FFM] and fat mass [FM]), direct measurements of blood compartment volumes (carbon monoxide [CO] rebreathing) and LV structure and function (echocardiography) in a group of carefully phenotyped middle‐aged individuals at IR for the development of HF. The primary aims of these analyses were to: (1) determine the impact of obesity class on blood compartment volumes (total blood, erythrocyte and plasma volumes), and (2) assess the relationship between blood compartment volumes and LV end‐diastolic volume (LVEDV). We hypothesized that Class I obesity would resemble non‐obese adults at IR after normalization of BV and LVEDV to body size, whereas Class II/III obesity would display distinct pathophysiology characterized by BV and LV expansion that is disproportionate to body size.

## Methods

### Ethical approval

Participants provided written informed consent. The protocol was approved by the Institutional Review Boards of the University of Texas Southwestern Medical Center (STU 062014–068, STU 122010–093, STU 062014–067, STU 032008–012). All studies were performed in accordance with the Declaration of Helsinki.

### Participants

This is a retrospective analysis of data combined from four clinical trials performed between 2012–2020 with common assessments of body composition, blood compartment volumes, and ventricular morphology. Participants are pooled from two cohorts: (1) sedentary healthy non‐obese adults (control, CON; *n* = 96), and (2) adults at increased risk with stage A or B HF (IR; *n* = 126).

Control participants were pooled from two studies (NCT02039154, *n* = 61; NCT01014572, *n* = 35) that enrolled sedentary, but otherwise healthy middle‐aged (45–64 years) participants recruited from the Dallas Heart Study, Cooper Center Longitudinal Study, or the local area.^16^, ^17^ Participants were excluded if they met the following criteria: BMI >30 kg/m^2^; exercise for ≥30 min three sessions/week; history of lung disease, hypertension, LV hypertrophy, coronary artery disease or structural heart disease.

Adults considered to be at IR were pooled from two studies (NCT03448185, *n* = 78; NCT03476785, *n* = 48).^18^, ^19^ Volunteers (age 40–64 years) were determined to be at IR by the presence of an elevated cardiac biomarker associated with elevated future risk of HF development (either N‐terminal pro‐B‐type natriuretic peptide [NT‐proBNP] >40 pg/ml or high‐sensitivity cardiac troponin T >0.6 pg/ml)^20^, ^21^, ^22^ and either (1) BMI >30 kg/m^2^ and visceral fat content >2 kg (dual‐energy x‐ray absorptiometry [DEXA]) or (2) LV hypertrophy by magnetic resonance imaging (>125 g/m^2^) or echocardiography (LV septum >11 mm). Participants at IR were separated by obesity class: Non‐obese: <30 kg/m^2^ (IR_Non‐Obese_), Class I: >30 and <35 kg/m^2^ (IR_Class‐I_), and Class II/III: >35 kg/m^2^ (IR_Class‐II/III_). Exclusion criteria included: signs or symptoms of HF; a history of ischaemic heart disease, myocardial infarction, stroke, greater than moderate valvular heart disease, atrial fibrillation, lung disease, sleep apnoea or insulin‐dependent diabetes; the inability to exercise.

### Experimental measures

#### Body composition

In the IR groups, FM and FFM were measured using DEXA (Lunar iDXA, GE Healthcare). In controls, FM and FFM were measured using underwater weighing (UWW) and calculated using age‐ and sex‐adjusted versions of the Siri equation.^23^, ^24^ Group estimates of %BF by UWW and DEXA have previously been shown to have high agreement and minimal bias for the analyses proposed in individuals <60 years of age.^24^, ^25^, ^26^, ^27^ Data from both are presented as two compartments: FM and FFM.

#### Blood compartment volumes

Carbon monoxide rebreathing^28^ was used to determine total haemoglobin mass (tHbmass) and blood, erythrocyte (EV) and plasma volumes (PV) (i.e. blood compartment volumes) using either the 20‐min CO rebreathing method, or the 2‐min “optimized” CO rebreathing method. There is no significant difference in tHbmass for eight healthy individuals assessed via both the 20‐ and 2‐min methods (20‐min: 713 ± 225 vs. 2‐min: 706 ± 251 g, *p* = 0.756).

#### Left ventricular structure, function and haemodynamics

Left ventricular volumes were assessed by three‐dimensional echocardiography (iE33; Phillips), with LVEDV analysed using Qlab 9.0 (Phillips) by an experienced blinded sonographer. The typical error of the LVEDV measurement in our laboratory is 10% (95% confidence interval [CI] 8–12%). Tissue and pulsed‐wave Doppler were also analysed (Qlab 9.0) to measure early diastolic and late diastolic filling velocities of the tissue (e') and blood (E and A), respectively. Peripheral blood pressure was measured via electrosphygmomanometry (Suntech Tango M2, NC). Central (ascending aortic) blood pressure was estimated using the SphygmoCor CVMS transfer function (AtCor Medical) applied to radial tonometric recordings calibrated against brachial systolic and diastolic pressure.

### Statistical analyses

To address aim 1, categorial data were compared using chi‐squared analysis while continuous data were compared using parametric (ANOVA) or non‐parametric (Kruskal–Wallis) tests dependent on normality testing (Shapiro–Wilk). We corrected for multiple comparisons using Tukey (parametric) or Benjamini and Hochberg (non‐parametric) post‐hoc tests. For aim 2, we used simple linear regression and multivariate linear regression to assess relationships between variables. Correlation coefficients were compared using the “cocor” (Comparing Correlations) package for R. Regression slopes were compared using ANOVA. We considered correlations to be either weak (*r* = 0.10–0.29), moderate (*r* = 0.30–0.49) or strong (*r* ≥ 0.5). Collinearity (tolerance) was assessed for all variables of interest in our multivariate regressions and all were above 0.1 indicating sufficiently low collinearity.^29^ Variables were chosen (1) based on the specific hypothesis we wanted to test regarding lean mass and FM, and (2) other variables known to be primary physiological determinants of BV (sex, age) or LVEDV (sex, age, mean arterial pressure). All statistical analyses were performed using R Studio (version 2023.06.2 + 561). Data are reported as *n* (percent), mean (standard deviation) or median [interquartile range]. For all analyses, *p* < 0.05 was considered statistically significant.

## Results

### Participant characteristics

The combined cohort of 222 adults were 52 ± 6 years of age, with a BMI 31.3 ± 7.3 kg/m^2^ and included 118 (53%) females. The IR_Class‐II/III_ group was younger than the two non‐obese groups (CON: 53 ± 6 years, IR_Non‐Obese_: 53 ± 5 years, IR_Class‐II/III_ 50 ± 6 years; *p* = 0.03 vs. both) but not compared to IR_Class‐I_ (51 ± 6 years; *p* = 0.55). The proportion of white individuals was greater in controls compared to all three IR groups (*p* < 0.001), with no difference between the three IR groups. Anthropomorphic variables are presented in *Table* *1*. All three IR groups demonstrated higher central and peripheral blood pressures compared to controls (*p* < 0.001) (*Table* *2*); there were no differences in hypertension medication treatment or blood pressure among IR groups (*p* = 0.17).

**Table 1:** Participant characteristics

Variable	Control (*n* = 96)	IR_Non‐Obese_ (*n* = 28)	IR_Class‐I_ (*n* = 39)	IR_Class‐II/III_ (*n* = 59)	*p*‐value
Female sex, %	54	43	40	63	0.25
Age, years	53 [47–57]	54 [51–56]	51 [47–55]	50 [44–54]* ^,^ ^†^	**0.029**
Race, %					**0.0046**
Caucasian	78	57	50	58
Black	5	39	24	32
Other	17	4	26	10
Anthropometric					
Height, cm	169 [164–177]	173 [165–179]	171 [163–176]	168 [163–173]	0.34
Weight, kg	73.0 [64.1–84.8]	81.8 [75.4–90.2]*	94.6 [87.1–104]* ^,^ ^†^	118 [104–131]* ^,^ ^†^ ^,^ ^‡^	**<0.0001**
BMI, kg/m^2^	25.8 [23.6–28.6]	28.2 [24.8–29.9]	33.0 [32.0–33.6]* ^,^ ^†^	39.7 [37.1–43.8]* ^,^ ^†^ ^,^ ^‡^	**<0.0001**
BSA, m^2^	1.9 (0.3)	2.0 (0.2)*	2.1 (0.3)*	2.4 (0.3)* ^,^ ^†^ ^,^ ^‡^	**<0.0001**
Body composition					
*n*	96	28	39	59	
Fat free mass, kg	47.5 [41.8–59.9]	53.9 [44.5–63.2]	56.2 [50.3–68.6]*	61.3 [54.5–67.8]* ^,^ ^†^	**<0.0001**
Fat mass, kg	25.1 [19.2–29.5]	29.1 [22.3–34.2]*	37.5 [35.7–42.3]* ^,^ †	52.1 [47.2–60.9]* ^,^ ^†^ ^,^ ^‡^	**<0.0001**
Body fat percent, %	31.3 [28.0–37.7]	33.4 [28.4–41.4]	40.2 [35.2–42.9]* ^,^ †	48.0 [43.2–50.8]* ^,^ ^†^ ^,^ ^‡^	**<0.0001**
Laboratory values					
*n*		25	37	54	
NT‐proBNP, pg/ml	–	49 [25–58]	37 [17–45]^†^	59 [31–88]^‡^	**0.001**
Medications					
Beta‐blocker	–	7 (25%)	7 (18%)	8 (14%)	
ACE inhibitor/ARB	–	13 (46%)	15 (38%)	18 (31%)	
CCB	–	6 (21%)	3 (8%)	11 (19%)	
Thiazide	–	5 (18%)	7 (18%)	7 (12%)	
Mineralocorticoid receptor antagonist	–	0	1 (3%)	3 (5%)	
Loop diuretic	–	0	0	1 (2%)	
HF stage, *n* (%)					
A	–	4 (14)	18 (46)	56 (95)	
B	–	24 (86)	21 (54)	3 (5)	

Data are presented as mean (standard deviation) or %, or median [interquartile range].

ACE, angiotensin‐converting enzyme; ARB, angiotensin receptor blocker; BMI, body mass index; BSA, body surface area; HF, heart failure; IR, increased risk; NT‐proBNP, N‐terminal pro‐B‐type natriuretic peptide.

* *p* < 0.05 vs. control.

^†^ *p* < 0.05 vs. IR_Non‐Obese_.

^‡^ *p* < 0.05 vs. IR_Class‐I_.

**Table 2:** Left ventricular structure and function, haemodynamics and haematology

Variable	Control (*n* = 96)	IR_Non‐Obese_ (*n* = 28)	IR_Class‐I_ (*n* = 39)	IR_Class‐II/III_ (*n* = 59)	*p*‐value
LV structure and function					
*n*	96	27	36	58	
Heart rate, bpm	80 (13)	80 (13)	84 (16)	85 (14)	0.13
LVEDV, ml	89 (21)	99 (22)*	113 (30)* ^,^ ^†^	136 (28)* ^,^ ^†^ ^,^ ^‡^	**<0.001**
LVEDV index, ml/m^2^	47.6 (8.9)	50.2 (10.1)	52.6 (12.4)*	58.4 (11.6)* ^,^ ^†^	**<0.001**
Mitral E wave^a^, cm/s	65 [57–74]	75 [65–85]*	69 [61–83]	80 [73–91]* ^,^ ^†^	**<0.001**
Mitral A wave^a^, cm/s	48 [43–55]	61 [57–69]*	58 [52–71]*	63 [49–70]*	**<0.001**
Mitral E/A wave, cm/s	1.37 [1.14–1.57]	1.19 [1.11–1.40]	1.26 [1.04–1.42]	1.29 [1.1–1.57]	0.39
e'	9.9 (1.8)	8.4 (1.9)*	8.8 (1.7)*	9.1 (1.8)*	**<0.001**
E/e'	6.75 [5.63–8.49]	8.76 [7.62–13.0]*	8.21 [7.22–10.2]*	8.89 [7.74–10.3]*	**<0.001**
Haemodynamics					
*n*	86	26	38	55	
Central SBP, mmHg	100 (120)	110 (10)*	109 (13)*	111 (12)*	**<0.001**
Central DBP, mmHg	63 [58–69]	73 [66–78]*	77 [69–80]*	73 [66–80]*	**<0.001**
Central MAP, mmHg	80 (10)	90 (9)*	90 (10)*	90 (9)*	**<0.001**
Brachial SBP, mmHg	114 [107–121]	126 [118–134]*	124 [106–134]*	118 [109–132]*	**<0.001**
Brachial DBP, mmHg	82 (8)	86 (9)	83 (10)	79 (12)^†^	**0.017**
Brachial MAP, mmHg	93 [87–98]	100 [96–104]*	96 [85–102]	92 [86–98]^†^	**0.003**
Haematology					
*n*	96	23	36	50	
Haemoglobin, g/dl	13.2 (1.3)	13.8 (1.6)	14.0 (1.6)*	13.5 (1.4)	0.02
Haematocrit, %	40 [37–42]	41 [38–44]	41 [38–45]	41 [39–44]	0.18
tHbmass, g	587 [482–803]	724 [543–847]	784 [604–903]*	777 [667–976]*	**<0.001**
tHbmass, g/kg	8.1 [7.3–9.4]	8.5 [8.0–9.4]	7.9 [6.7–8.8]	6.7 [6.2–7.6]* ^,^ ^†^ ^,^ ^‡^	**<0.001**
tHbmass, g/kg FFM	12.5 (1.5)	12.8 (1.7)	13.0 (2.2)	13.1 (1.9)	0.31
BV, ml/kg FFM	100.6 [93.4–108.0]	101.8 [98.2–107.7]	102.2 [91.5–114.0]	105.4 [100.1–109.4]	0.160
EV, ml/kg FFM	36.2 (4.6)	38.2 (4.6)	38.8 (6.1)	39.9 (5.1)*	**<0.001**
PV, ml/kg FFM	63.3 [59.5–70.4]	64.5 [60.0–66.5]	61.9 [55.0–72.4]	66.6 [60.5–72.2]	0.466

Data are presented as mean (standard deviation), or median [interquartile range].

BV, blood volume; DBP, diastolic blood pressure; EV, erythrocyte volume; FFM, fat free mass; LV, left ventricular; LVEDV, left ventricular end‐diastolic volume; MAP, mean arterial pressure; PV, plasma volume; SBP, systolic blood pressure; tHbmass, total haemoglobin mass.

^a^ Mitral E and A for the control group have 82 and 80 participants, respectively.

* *p* < 0.05 vs. control.

^†^ *p* < 0.05 vs. IR_Non‐Obese_.

^‡^ *p* < 0.05 vs. IR_Class‐I_.

### Blood compartment volumes

**Figure 1:**
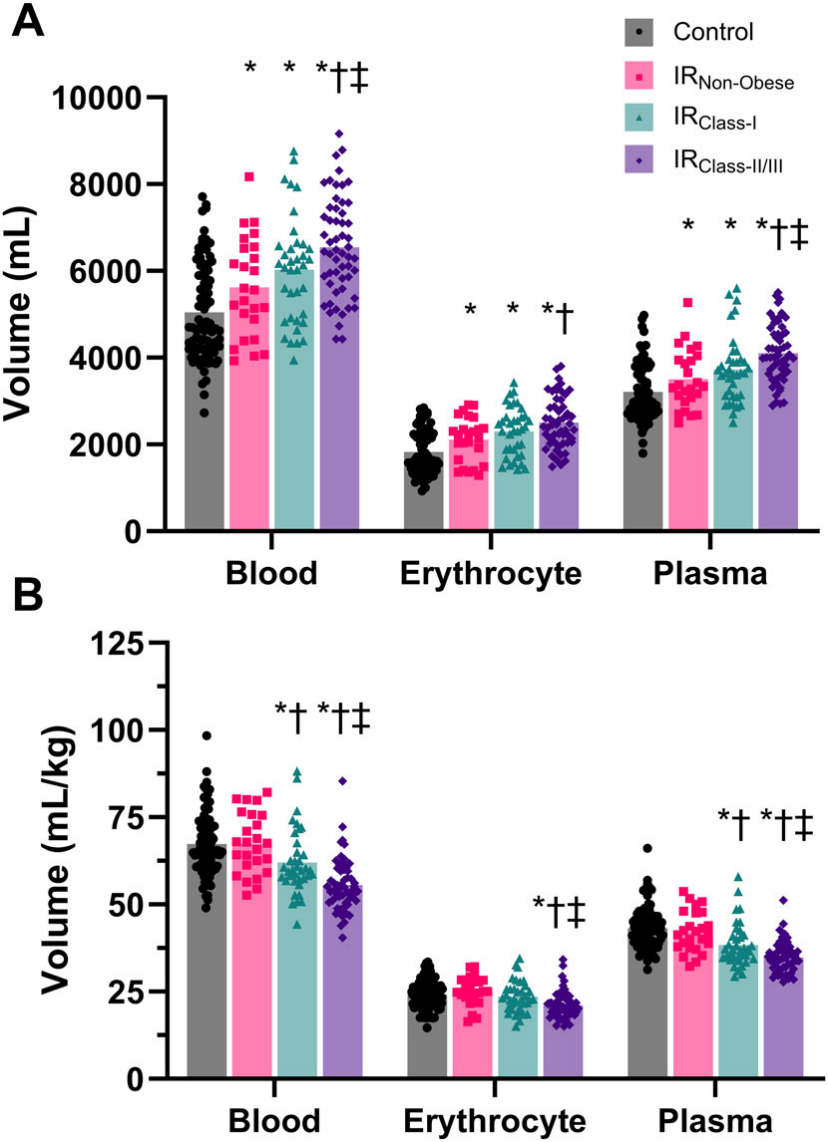
Blood compartment volumes in controls and middle‐aged adults at increased risk. (*A*) Absolute blood volume (BV), erythrocyte volume (EV), and plasma volume (PV) in controls (CON, *n* = 89), increased risk non‐obese (IR_Non‐Obese_; *n* = 25), increased risk Class I obesity (IR_Class‐I_; *n* = 37) and increased risk Class II/III obesity (IR_Class‐II/III_; *n* = 55). (*B*) BV, EV, and PV relative to body mass. *p < 0.05 vs. CON; ^†^ *p* < 0.05 vs. IR_Non‐obese_; ^‡^ *p* < 0.05 vs. IR_Class‐I_.

Absolute BV was higher in all IR groups compared to controls (all *p* < 0.05) (*Figure* *1*). BV was greater in IR_Class‐II/III_ than both other IR groups (IR_Non‐Obese_: 5622 ± 1137 ml, IR_Class‐I_: 6033 ± 1237 ml, IR_Class‐II/III_: 6548 ± 1153 ml, all *p* < 0.05) (*Figure* *1A*). EV and PV were larger in IR_Class‐II/III_ compared to controls and the other IR groups (all *p* < 0.02). When scaled to body mass, BV was lower in IR_Class‐II/III_ compared to all other groups (CON: 67.3 [62.8–73.8] ml/kg, IR_Non‐Obese_: 67.7 [60.0–75.8] ml/kg, IR_Class‐I_: 60.0 [57.1–67.7] ml/kg, IR_Class‐II/III_: 54.9 [50.6–59.9] ml/kg; *p* < 0.05), as was PV and EV scaled to body mass (*Figure* *1B*). Absolute tHbmass was higher in the obese IR groups compared to controls (both *p* < 0.003), but when scaled to body mass was lower in IR_Class‐II/II_ compared to all other groups (*p* < 0.01) (*Table* *2*). After normalization of compartment volumes to FFM there was no significant difference in BV between groups (*p* = 0.160), but EV remained significantly elevated in IR_Class‐II/II_ compared to controls (CON: 36.2 ± 4.6 ml/kg FFM, IR_Class‐II/III_: 39.9 ± 5.1 ml/kg FFM; *p* < 0.001).

### Left ventricular structure and function

**Figure 2:**
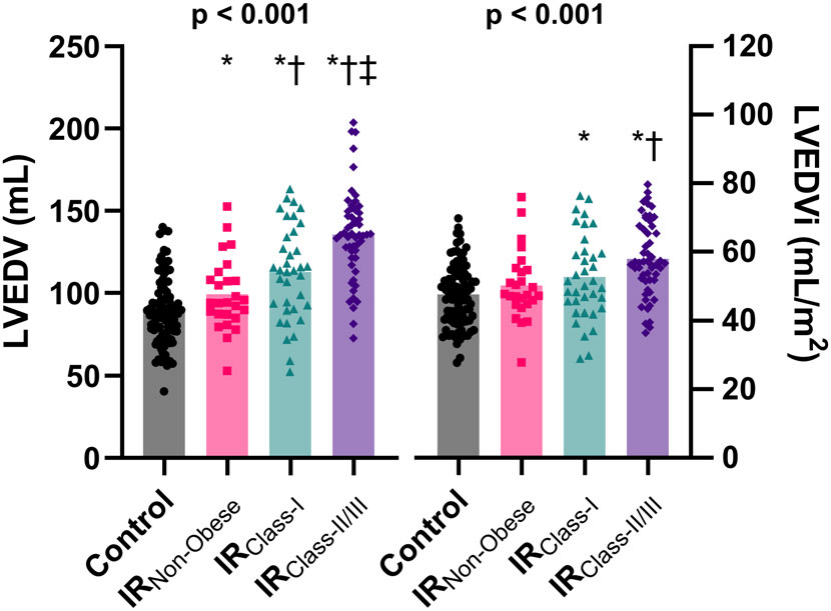
Left ventricular end‐diastolic volume (LVEDV) in controls and middle‐aged adults at increased risk. Absolute LVEDV (left panel) and LVEDV index (LVEDVi) (right panel) in controls (CON, *n* = 96), increased risk non‐obese (IR_Non‐Obese_; *n* = 26), increased risk Class I obesity (IR_Class‐I_; *n* = 36) and increased risk Class II/III obesity (IR_Class‐II/III_; *n* = 54). *p < 0.05 vs. CON, ^†^ *p* < 0.05 vs. IR_Non‐obese_; ^‡^ *p* < 0.05 vs. IR_Class‐I_.

Absolute LVEDV was highest in IR_Class‐II/III_ and was larger in all IR groups compared to controls (*p* < 0.001) (*Figure* *2*). When indexed to body surface area, LVEDV index (LVEDVi) was not different between controls (47.6 ± 8.9 ml/m^2^) and IR_Non‐Obese_ (50.2 ± 10.1 ml/m^2^) or between IR_Non‐Obese_ and IR_Class‐I_ (52.6 ± 12.4 ml/m^2^; both *p* > 0.99), whereas IR_Class‐II/III_ had a larger LVEDVi (58.0 ± 10.9 ml/m^2^) compared to controls (*p* < 0.01), IR_Non‐Obese_ (*p* = 0.01) and IR_Class‐I_ (*p* = 0.09). Mitral E wave differed between controls and the IR_Non‐Obese_ (*p* = 0.04), IR_Class‐II/III_ (*p* = 0.04) groups (*Table* *2*), while mitral late diastolic filling velocity (A) was higher in the IR groups when compared to controls (all *p* < 0.02). There was no difference in E/A ratio between groups (*p* = 0.39). E/e' was higher in all IR groups compared to controls (*Table* *2*) but was not different among the IR groups.

### The association of fat‐free mass and fat mass with blood volume

**Figure 3:**
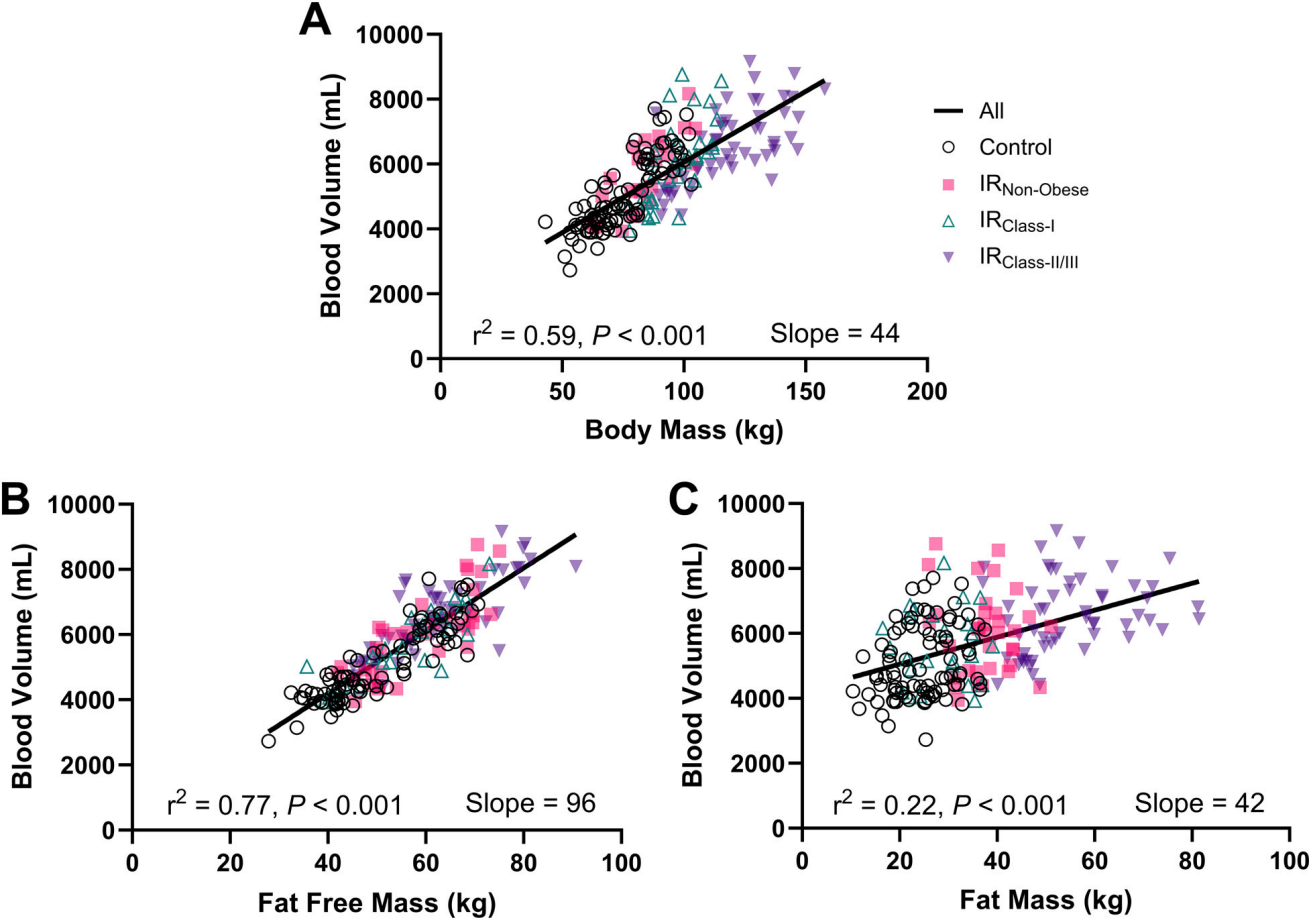
Linear relationship between body mass, fat‐free mass and fat mass and blood volume in controls and middle‐aged adults at increased risk. Linear relationship between blood volume and (*A*) body mass, (*B*) fat‐free mass, and (*C*) fat mass. *R* ^2^ and slope are derived from all groups combined.

With all data merged, there was a strong positive linear correlation between body mass and BV (*r* = 0.77, *p* < 0.001; slope = 44 ml/kg) (*Figure* *3*). FFM had a stronger correlation with BV (*r* = 0.84, *p* < 0.001, slope = 96 ml/kg) than total body mass (*p* < 0.001, for comparison of *r* values) and FM (*r* = 0.47, *p* < 0.001, slope = 42 ml/kg; *p* < 0.001). Linear regressions of BV and body FFM and FM are shown in online supplementary *Figures* *S1*–*S3*. Univariate regression indicated that FFM, FM, and sex contribute to the variation in BV, while multivariate regression indicated that FM was associated with higher BV (*β* = 0.16, *p* < 0.001) independent of FFM (*β* = 0.76, *p* < 0.001), age, and sex (*Table* *3*).

**Table 3:** Multiple linear regression for association of blood volume and ventricular end‐diastolic volume

Covariates	Blood volume, sqrt			
	Univariate		Multivariate (r^2^ = 0.79)	
	*β*	*p*‐value	*β*	*p*‐value
Fat free mass, sqrt	0.88	**<0.001**	0.76	**<0.001**
Fat mass, log	0.49	**<0.001**	0.16	**0.001**
Age	−0.03	0.698	0.05	0.151
Male sex	6.43	**<0.001**	0.07	0.315

	LVEDV: Model 1, log			
	Univariate		Multivariate (*r* ^2^ = 0.55)	
Blood volume, sqrt	0.71	**<0.001**	0.54	**<0.001**
Fat free mass, sqrt	0.62	**<0.001**	0.09	0.482
Fat mass, log	0.55	**<0.001**	0.24	**0.001**
Age	−0.13	0.070	−0.08	0.109
Male sex	0.31	**<0.001**	−0.08	0.390
Mean arterial pressure	0.249	**<0.001**	−0.05	0.356

	LVEDV: Model 2, log			
	Univariate		Multivariate (*r* ^2^ = 0.57)	
Blood volume, sqrt	0.71	**<0.001**	0.52	**<0.001**
Body mass index, log	0.64	**<0.001**	0.32	**<0.001**
Age	−0.13	0.070	−0.07	0.152
Male sex	0.31	**<0.001**	−0.020	0.776
Mean arterial pressure	0.249	**<0.001**	−0.03	0.555

LVEDV, left ventricular end‐diastolic volume, sqrt, square root.

### The association of blood volume with left ventricular size

**Figure 4:**
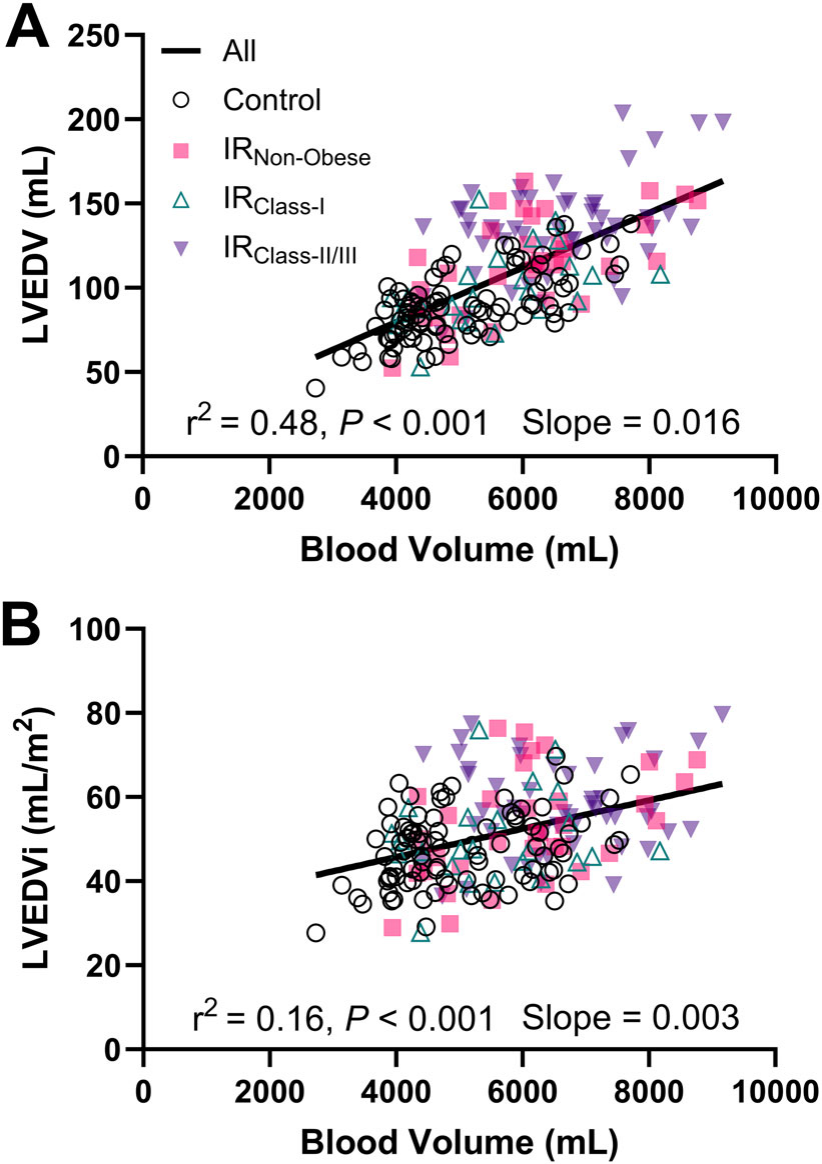
Linear relationship between blood volume and left ventricular end‐diastolic volume (LVEDV) in controls and middle‐aged adults at increased risk. (*A*) LVEDV and (*B*) LVEDV index (LVEDVi). *R* ^2^ and slope are derived from all groups combined.

Across all participants, BV was positively correlated with absolute LVEDV (*r* = 0.66, *p* < 0.001; slope = 0.016) and LVEDVi (*r* = 0.4, *p* < 0.001; slope = 0.0033) (*Figure* *4*). Linear regressions for each group are shown in online supplementary *Figures* *S4* and *S5*. As assessed via univariate regression, BV, FFM, and FM positively correlated with LVEDV, whereas male sex was associated with larger LVEDV (*Table* *3*). Using multivariate regression, BV (*β* = 0.54, *p* < 0.001) and FM (*β* = 0.24, *p* < 0.001) were independently associated with LVEDV (*Table* *3*). Because BMI is derived from FM and FFM, we assessed the relationship between BMI and LVEDV in a separate model and identified that BMI is associated with LVEDV (*β* = 0.32, *p* < 0.001), independent of BV (*β* = 0.52, *p* < 0.001), and age, sex, and mean arterial pressure (*Table* *3*).

## Discussion

The key findings of this study, the largest to date with measured BV and LV morphology in adults with biomarker‐confirmed IR for HF, were that both non‐obese and obese adults at IR have expanded absolute blood compartment and ventricular volumes relative to non‐obese controls that was graded with the severity of obesity. However, IR_Class‐II/III_ obesity displayed alterations in blood compartment volumes and LV remodelling that were disproportionate to body size and distinct from IR_Class‐I_ and IR_Non‐Obese_. The most prominent alterations in IR_Class‐II/III_ were a larger LVEDVi and smaller EV per body mass compared to IR_Non‐Obese_ and controls. We report that FM is independently associated with BV expansion, and that BV and FM are independently associated with LVEDV. These results have important implications for understanding of the progression from IR pre‐HF in middle age to HFpEF in older age, and for understanding potential differential responsiveness of Class II/III obesity to therapeutic interventions.

### Adiposity is associated with blood volume expansion in increased risk adults

Blood volume regulation in humans involves several organ systems and signalling pathways. Obesity leads to an expansion of BV that is considered to be in proportion to body size to accommodate increases in capillarization and metabolic demand of expanded muscle mass and adipose tissue.^15^ While often posited that FM, either subcutaneous or visceral, provides a stimulus for BV expansion it is difficult to separate the effects of body size versus composition. Few studies have measurements of body composition and BV spanning a wide range of BMI, and many investigations consider obesity broadly as opposed to by class. In this cohort of highly phenotyped middle‐aged adults at IR and controls, we confirm previous reports that FFM is a primary determinant of BV, and extend these findings to show an independent role of FM in BV expansion. The weaker association between FM and BV likely arises from higher capillary density and metabolic capacity of fat‐free tissue relative to adipose. The independent association between adiposity and BV expansion is a critical link supporting the hypothesized role of adipose‐mediated BV expansion and the progression of HF in obesity that had previously been unreported. The mechanisms that mediate the association between increased adiposity and BV expansion are not well defined but may include metabolic pressure from expanding adipose to support perfusion of adipose, or direct actions of adipose tissue on natriuretic peptide metabolism and renin–angiotensin–aldosterone system signalling.

### Concurrent hyper‐ and hypovolaemia in increased risk adults with obesity

The relative importance of absolute hypervolaemia versus relative hypovolaemia in the progression of HF is unclear and each may contribute independently. All groups at IR in this cohort had expanded absolute blood compartment volumes compared to similarly aged male and female adults, including IR_Non‐Obese_ adults. The absolute hypervolaemia remained in both obese groups at IR compared to controls after normalization to FFM,^30^ and IR_Class‐II/II_ showed a distinctly higher EV normalized to lean mass. In lean individuals, adipose tissue is not considered to contribute substantially to EV regulation due to its small mass and low metabolic demand. However, the increase in erythrocyte production in Class II/III indicates that collective metabolic demand of excess adipose, or adipose associated renin–angiotensin signalling may create a sufficient signal to increase EV. The expanded absolute BV likely impacts the heart where chronic volume stress is hypothesized to induce dilated remodelling and cardiac growth, with or without ventricular thickening.^31^ In our cohort both non‐obese and obese IR adults were selected based upon the presence of biomarkers that associate with elevated cardiac wall stress (NT‐proBNP), low level cardiac injury (cardiac troponin T), and HF risk.^31^ Importantly, we show a distinct synergy between the presence of these cardiac biomarkers and severe obesity whereby IR_Class‐II/III_ had LVEDV remodelling in excess of that observed in controls and IR_Non‐Obese_.^31^ Importantly, we show a distinct synergy between the presence of these cardiac biomarkers and severe obesity whereby IR_Class‐II/III_ had LVEDV remodelling in excess of that observed in controls and IR_Non‐Obese_. These findings support the hypothesis that circulating hypervolaemia serves as a key stimulus for LV remodelling especially in adults at IR with severe obesity, potentially contributing to the progression HFpEF and high‐output HF in advanced obesity.^32^

In contrast to the observed absolute hypervolaemia, normalization of blood compartment volumes to body mass indicates that IR_Class‐I_ and IR_Class‐II/III_ are characterized by a relative hypovolaemia that is markedly lower in IR_Class‐II/III_. The decrease in blood compartment volumes per body mass is consistent with decreased capillary density of tissues in adults with obesity including skeletal muscle, subcutaneous and visceral fat depots, renal and cardiac tissue.^33^ Relative hypovolaemia may contribute to end‐organ dysfunction via several mechanisms including poor tissue perfusion, renin–angiotensin system activation, and tissue hypoxia and inflammation.^12^, ^34^ Diminished relative oxygen carrying capacity coupled with greater intrinsic metabolic demand for activities of daily living due to excess body weight may by key contributors to the development of exercise intolerance in severely obese adults. Finally, low relative blood compartment volumes could be especially important when considering obesity in the larger picture of its comorbid conditions and treatments that impact the ability of an individual to maintain proportional volume status including type II diabetes, chronic kidney disease, and therapeutic diuresis.^34^, ^35^

### Left ventricular remodelling and future heart failure in obese adults at increased risk

Recently, it has been hypothesized that cardiac atrophy due to a lack of physical activity is a key risk factor contributing to the development of HFpEF in certain individuals,^36^ and that the higher prevalence of HFpEF in women is explained by smaller hearts being more susceptible to having a higher filling pressure.^37^ While these hypotheses may be true for the small proportion of lean adults with HFpEF, our data suggest a more complicated relationship between physical activity, heart size, and HFpEF progression for the majority (up to 80%) of adults with HFpEF and obesity. In our cohort of adults at IR, the absolute LVEDV in IR_Class‐II/III_ was 25% larger than controls even after normalization to body mass, and strikingly similar to the LVEDV achieved following 2 years of intensive aerobic exercise training in a subset of the control group studied here (~58 ml/m^2^).^17^ Further, 31 of the 37 women in the IR_Class‐II/II_ group have unindexed LVEDVs above the average value for healthy controls, arguing against cardiac atrophy secondary to deconditioning as a primary mechanism underlying the development of HFpEF in severely obese adults. While increasing physical activity is associated with lower risk of HFpEF,^38^ the primary mechanism by which physical activity protects against HFpEF may have more to do with pleiotropic effects of exercise on metabolic function and cardiac stiffness and function as opposed to LV remodelling, as the heart of severely obese IR middle‐aged adults is in fact quite large.

Therefore, we hypothesize that the marked increase in skeletal muscle mass, LVEDV, and blood compartment volumes in IR middle‐aged adults with severe obesity may in part represent an adaptive circulatory response to the chronic physical demand of carrying significant levels of excess body weight. The residual adaptive capacity of the cardiovascular system in middle age prior to the onset of age‐associated stiffness and cardiac atrophy,^16^ may in part mask the traditional ‘sedentary‐small heart phenotype’. While on the surface these adaptations mirror some of the hallmark adaptations of athletes in middle age, they are more susceptible to the key pathophysiological consequences of ageing (i.e. vascular and cardiac stiffening), and metabolic syndrome (decreased muscle oxidative capacity) which eventually lead to the cardinal manifestation of HFpEF: exercise intolerance. Further work is needed to determine how obesity and ageing interact to influence cardiac function and peak aerobic capacity in adults at IR and identify interventions in middle age prior to the acceleration of age‐associated cardiovascular dysfunction.^19^ Additionally, an important area of future investigation is the interaction between pharmacologic weight loss which reduces FM and FFM, key independent predictors of LVEDV, and concurrent exercise training which increases LVEDV in severely obese adults.^19^ More work is needed to develop exercise strategies that are compatible with pharmacologic weight loss, reduce skeletal muscle wasting and preserve cardiac size and function.

### Clinical implications

Our study suggests that adults at IR with Class II/III obesity are physiologically distinct from Class I obese and non‐obese adults at IR. The unique phenotype of Class II/III obesity in middle age may provide important insight regarding the pathogenesis of the increasingly prevalent ‘obese phenotype’ of HFpEF which is characterized by large heart volumes, ventricular interdependence, increased BV, exaggerated cardiac output responses to exercise, and poor skeletal muscle oxidative capacity, but not mass.^11^ As weight loss medications continue to revolutionize the treatment of obesity‐associated disorders, clinical trials with sufficient power to consider severe obesity independent of overweight and Class I obesity are needed as the efficacy of these treatments on key cardiovascular outcomes may vary by obesity class. The knowledge gap left by systematic underrepresentation of adults with Class II/III obesity in clinical trials of HFpEF therapies^6^ provides an opportunity to better elucidate the pathophysiological consequences of advanced obesity and its management. Given the unique importance of BV to the pathophysiology of severe obesity, and the poor performance of calculated estimates of blood compartment volumes in patients with HF,^13^ future clinical investigations and interventions utilizing direct measures of blood compartment volumes are needed to improve understanding and management of BV status in severely obese patient populations. Finally, the presence of a large left ventricle in some IR adults with Class II/III obesity should not deter the use of exercise training as a primary lifestyle intervention reduce the effects of cardiac ageing.^17^ In this cohort of adults at IR and obesity, 1‐year of high intensity interval training improved LV function and functional capacity with no change in concentricity index.^19^

### Study limitations

We determined adults to be at IR based on elevated cardiac biomarkers and either LV hypertrophy or severe obesity. Therefore, our IR_Non‐Obese_ comparator group is by necessity comprised only of individuals who qualified based upon LV hypertrophy, whereas our IR groups with obesity represent a mix of individuals who qualified with and without LV hypertrophy (percentage with LV hypertrophy: IR_Class‐I_: 64% and IR_Class‐II/III_: 50%). Our selection bias most directly impacts interpretation of comparisons of LVEDV made with the IR_Non‐Obese_ group which may have greater representation of individuals with concentric LV remodelling that, if severe enough, would decrease LVEDV. However, the IR_Non‐Obese_ had a larger LVEDV compared to controls, and similar LVEDVi compared to IR_Class‐I_, arguing against any systematic reduction in chamber size. Nevertheless, the key conclusions regarding LV remodelling in IR_Class‐II/III_ remain unchanged when controls or IR_Class‐I_ are the comparator group. Because obesity is a specific and stronger predictor of future HFpEF than HF with reduced ejection fraction,^2^, ^3^, ^4^ we frame the rationale and discussion around the pathophysiology of HFpEF. However, due to the cross‐sectional nature of the investigation we are unable to make any conclusions regarding the role of BV dysregulation and the risk for future HFpEF.

## Conclusion

While lean mass is the primary determinant of BV, our data provide evidence for an independent association between FM, BV expansion and LV remodelling in middle‐aged adults at IR for developing HF (*Graphical Abstract*). Importantly, in adults at IR for HF, Class II/III obesity was associated with LV remodelling that was physiologically distinct from their Class I and non‐obese IR counterparts. Increased risk Class II/III obesity is characterized by the coexistence of absolute hypervolaemia, relative hypovolaemia, and marked LV enlargement that is disproportionate to body size. The marked expansion of FFM, blood compartment volumes, and LV remodelling in middle‐aged adults at IR may be an adaptation to the physical demands of extreme obesity which may provide insight to the pathogenesis of the obese HF phenotype.

## Supporting information


**Appendix S1.** Supporting Information.
