# Supplementary material for: Distinct blood volume and left ventricular adaptation to severe obesity in middle‐aged adults at risk for heart failure
Source: Eur J Heart Fail. 2025 Sep 9;27(11):2480–9. doi: 10.1002/ejhf.70037 (PMC12765370; doi:10.1002/ejhf.70037)
Supplement: Supplementary file 1 — Appendix S1. Supporting Information. [file EJHF-27-2480-s001.zip › ejhf70037-sup-0001-Captions.docx]

**Supplementary Material**

**Figure S1. Linear relationship between body mass and blood volume in controls and middle-aged adults at increased risk.** Linear relationship between blood volume and body mass for controls (Panel A), increased risk non-obese (Panel B), increased risk class I obesity (Panel C) and increased risk class II/III obesity (Panel D). R^2^ and slope are derived for each group separately.

**Figure S2. Linear relationship between fat free mass and blood volume in controls and middle-aged adults at increased risk.** Linear relationship between blood volume and fat free mass for controls (Panel A), increased risk non-obese (Panel B), increased risk class I obesity (Panel C) and increased risk class II/III obesity (Panel D). R^2^ and slope are derived for each group separately.

**Figure S3. Linear relationship between fat mass and blood volume in controls and middle-aged adults at increased risk.** Linear relationship between blood volume and fat mass for controls (Panel A), increased risk non-obese (Panel B), increased risk class I obesity (Panel C) and increased risk class II/III obesity (Panel D). R^2^ and slope are derived for each group separately.

**Figure S4.** **Linear relationship between blood volume and left ventricular end diastolic volume (LVEDV) in controls and middle-aged adults at increased risk.** Linear relationship between blood volume and LVEDV for controls (Panel A), increased risk non-obese (Panel B), increased risk class I obesity (Panel C) and increased risk class II/III obesity (Panel D). R^2^ and slope are derived for each group separately.

**Figure S5.** **Linear relationship between blood volume and left ventricular end diastolic volume indexed to body surface area (LVEDVi) in controls and middle-aged adults at increased risk.** Linear relationship between blood volume and LVEDVi for controls (Panel A), increased risk non-obese (Panel B), increased risk class I obesity (Panel C) and increased risk class II/III besity (Panel D). R^2^ and slope are derived for each group separately.s
